# Severity of Intracranial Arterial Calcification on Computed Tomography and Risk of Dementia in Patients With Stroke or Transient Ischemic Attack: A Population‐Based Study

**DOI:** 10.1161/JAHA.125.046801

**Published:** 2026-03-04

**Authors:** Ke Li, Davide Simonato, Peter M. Rothwell

**Affiliations:** ^1^ Wolfson Centre for Prevention of Stroke and Dementia, Nuffield Department of Clinical Neurosciences University of Oxford Oxford UK

**Keywords:** dementia, intracranial arterial calcification, stroke, Vascular Disease, Transient Ischemic Attack (TIA), Cognitive Impairment, Cerebrovascular Disease/Stroke, Computerized Tomography (CT)

## Abstract

**Background:**

Coronary arterial calcification predicts coronary events, but although intracranial arterial calcification on CT (CT‐IAC) is a frequent finding in older individuals, few longitudinal studies have assessed whether its severity or site predict dementia. We did a population‐based study in patients with transient ischemic attack (TIA) or stroke to assess these associations.

**Methods:**

In a matched case–control study of patients with minor stroke/transient ischemic attack nested in the population‐based OXVASC (Oxford Vascular Study), severity (qualitatively and semiautomated volume) and location (intimal or internal elastic lamina) of CT‐IAC in cases who developed dementia on follow‐up was compared with that in age‐/sex‐matched controls who did not (logistic regression adjusted for other risk factors).

**Results:**

In OXVASC (cases/controls=200/200; mean age=78.0±9.3 years), dementia was independently associated with severity of internal carotid artery CT‐IAC on visual assessment (bilateral severe—adjusted OR [aOR], 2.02 [95% CI, 1.26–3.23], *P*=0.004) and quantitative volume (top versus bottom tertile—aOR, 2.35 [95% CI, 1.33–4.16], *P*=0.003), driven mainly by individuals with very high calcification volumes (≥600 mm^3^ versus 0–299 mm^3^—aOR, 6.23 [95% CI, 1.24–31.24], *P*=0.026). Similar trends were observed for CT‐IAC in the internal carotid artery and vertebrobasilar artery combined (top versus bottom tertile—aOR, 2.59[95% CI, 1.43–4.68], *P*=0.002), including after exclusion of recurrent stroke (aOR, 2.60 [95% CI, 1.33–5.08], *P*=0.005) and patients with moderate/severe white matter disease (aOR, 3.19 [95% CI, 1.54–6.62], *P*=0.002). Internal carotid artery CT‐IAC of the internal elastic lamina independently predicted dementia after adjusting for qualitative (aOR, 1.84 [95% CI, 1.11–3.05, *P*=0.019) or quantitative (aOR, 1.78 [95% CI, 1.06–2.99], *P*=0.029) CT‐IAC severity.

**Conclusions:**

Severity of CT‐IAC independently predicts future dementia after stroke/ transient ischemic attack. The extent of any nonlinearity and calcification‐ or dementia‐subtype differences should be determined in larger studies.

**Nonstandard Abbreviations and Acronyms**
CT‐IAC: computed tomography‐visualized intracranial arterial calcification
IEL: internal elastic lamina
OXVASC: Oxford Vascular Study

**Clinical Perspective**

**What Is New?**

- In a matched case–control study nested within the Oxford Vascular Study, we showed that severity of computed tomography (CT)‐visualized intracranial arterial calcification (CT‐IAC) independently predicted dementia risk during follow‐up in patients with stroke or transient ischemic attack and that the association was driven by a small number of patients with highest CT‐IAC.
- The apparent nonlinearity of the association and the particular predictive value of medial/internal elastic lamina CT‐IAC suggest that factors other than atherosclerosis are likely to explain the association.

**What Are the Clinical Implications?**

- Given that dementia risk is highest in patients with severe CT‐IAC, simple visual scales should be sufficient to identify at risk individuals in routine practice, and severe CT‐IAC could be noted on radiology reports and integrated into dementia prediction models.

Stroke and dementia are the most prevalent disabling neurological disorders in both high‐income and low‐income countries.^1^ The 2 conditions often coexist, each increasing the risk of the other, and they share several risk factors.^2^ Both conditions are also strongly age related, due partly to associations with vascular pathologies, including atherosclerosis and small vessel disease.^3^ Arterial calcification also increases with age, and coronary artery calcification has been shown in multiple studies to be a strong independent risk factor for acute coronary events,^4^, ^5^ with possible associations with stroke and dementia.^6^, ^7^

Calcification of the intracranial arteries is also a frequent finding in older individuals undergoing computed tomography (CT) brain imaging. Because the progression of arterial calcification is potentially reduced by medical treatments,^8^ it is important to understand any associations between calcification of the intracranial arteries and neurological disorders, particularly stroke and dementia. Although CT‐visualized intracranial arterial calcification (CT‐IAC) has been shown to be associated with an increased risk of stroke,^9^ the evidence for prediction of dementia is less established.

Some cross‐sectional studies have shown an association between CT‐IAC and coexisting cognitive decline or dementia,^10^, ^11^, ^12^ but the predictive value of CT‐IAC for future risk of dementia independent of other vascular risk factors has been reported in only a few publications from 2 longitudinal cohorts,^13^, ^14^, ^15^, ^16^ the first reports from the general population Rotterdam Study and the second in patients with transient ischemic attack (TIA) or stroke, although another longitudinal cohort that reported only univariable results showed null effect of CT‐IAC on dementia.^17^ Both cohorts with adjusted estimates suggested that severity of CT‐IAC was an independent predictor of dementia, albeit with borderline statistical significance,^13^, ^16^ and the Rotterdam Study also analyzed associations with coronary, aortic, and extracranial carotid artery calcification.^14^ However, in cross‐sectional^10^, ^11^, ^12^, ^18^, ^19^, ^20^ and longitudinal^13^, ^14^, ^15^, ^16^ studies on CT‐IAC and dementia, none have compared the predictive values of qualitative visual scales for presence and severity of CT‐IAC with quantitative semiautomated calcification measurements. Moreover, only the Rotterdam Study differentiated intimal versus medial CT‐IAC, a distinction that may have mechanistic implications.^13^ Calcification of the arterial intima is usually related to atheroma and inflammation and lipid deposition may therefore play a role.^8^, ^13^, ^16^, ^21^, ^22^ Calcification of the media and internal elastic lamina (IEL) is more often associated with metabolic disorders including diabetes, chronic kidney disease, and parathyroid disorders and is correlated with increased arterial stiffness and white matter hyperintensities, and hence possibly with risk of dementia.^8^, ^13^, ^21^, ^22^

Given the potential importance of CT‐IAC as a possibly treatable risk factor for dementia, we determined whether the severity of CT‐IAC predicts dementia independently of other established risk factors in a population‐based study of patients with TIA or stroke. First, we aimed to validate the findings of the Rotterdam Study and to increase the evidence‐base in patients with TIA or stroke. Second, we aimed to determine whether any association with dementia risk in patients with TIA and stroke was independent of recurrent stroke and of initial severity of stroke. Third, in terms of applicability in routine clinical practice, we aimed to compare the assessment and predictive value of CT‐IAC assessed using a simple visual scale versus a semiautomated quantitative scale. Finally, because coronary artery calcification is usually related to atherosclerotic plaque,^23^ whereas calcification in the aorta and intracranial ICA more often involves the media/IEL,^13^, ^24^ and calcification of the latter sites was shown to be more strongly associated with dementia than coronary calcification,^14^, ^25^ we aimed to determine whether the medial/IEL subtype of CT‐IAC was more strongly related to dementia than the intimal type.

## METHODS

Reasonable request for data supporting this study's findings will be considered by the chief investigator. For further details, please contact Professor Peter Rothwell (peter.rothwell@ndcn.ox.ac.uk).

### Study Population

To investigate the association between CT‐IAC and dementia following stroke/TIA, we conducted a longitudinal matched case–control analysis nested within the OXVASC (Oxford Vascular Study). OXVASC is a prospective, population‐based cohort study that investigates the incidence and outcomes of all acute vascular events in Oxfordshire, United Kingdom.^26^, ^27^ Detailed descriptions of the study population and methodology have been published previously.^26^, ^27^, ^28^ Briefly, OXVASC covers a population of individuals registered with 9 primary care practices (roughly 100 primary care doctors; midstudy population 94 567) in Oxfordshire, UK. Based on 2011 census data, 6.4% of the total resident population of Oxfordshire and 36.4% of Oxford city's population was from an ethnic minority (non‐“White‐British”) background, compared with 19.5% across England and Wales.^26^, ^27^, ^28^

Ascertainment of cases involved multiple overlapping methods in both the community and all health care settings of the study population.^26^, ^27^, ^28^ All participating primary care doctors were requested to refer patients who presented with new sudden onset transient neurological symptoms to a daily study‐specific TIA and stroke clinic, which provided urgent clinical investigation and treatment. Multiple other sources were used to achieve near‐complete prospective identification of patients presenting to medical attention with any possible vascular events: (1) daily visits to the emergency department of the single referral hospital in the region; (2) daily visits to the hospital's acute stroke unit, neurology wards, and coronary care unit; (3) daily visits to bereavement officers; (4) regular searches of hospital computerized diagnostic records for patients with symptoms of vascular events; (5) regular searches of records of requests for brain or neurovascular imaging; (6) daily searches of lists of all patients in whom a troponin‐I level had been requested; snf (7) monthly searches of primary care computer records for vascular diagnoses. The study received approval from the regional ethics committee (OREC A: 05/Q1604/70), and written informed consent was obtained from all patients, or assent was obtained from relatives of patients with dementia or dysphasia.

TIA was defined according to the National Institute of Neurological Disorders and Stroke criteria, and stroke was defined based on World Health Organization criteria.^26^, ^27^ Stroke severity was assessed using the National Institutes of Health Stroke Scale score.^26^, ^27^ Cognitive function was evaluated using the Mini‐Mental State Examination and Montreal Cognitive Assessment for face‐to‐face interviews,^29^ and the telephone versions of Montreal Cognitive Assessment and the modified Telephone Interview of Cognitive Status for remote assessments, both of which are validated for TIA/stroke.^30^ With multiple methods, follow‐up to end point was completed in >95% of patients.^31^, ^32^

Dementia following stroke/TIA was defined as that developed after the index cerebrovascular event, based on the *Diagnostic and Statistical Manual of Mental Disorders, Fourth Edition* criteria.^3^ Dementia diagnoses were informed by clinical and cognitive assessments and informant reports, supplemented by reviews of primary care records, including clinic letters, hospital records, consultations, and death certificates. All diagnoses were made by a senior study physician with expertise in dementia.^3^

The sample size calculation (G*Power 3.1.9.7) (http://www.gpower.hhu.de) for the case–control study was designed to allow validation of the association of calcification severity with dementia risk observed in the 2 previous longitudinal studies.^13^, ^16^ A sample of 200 cases and 200 age‐/sex‐matched controls afforded 80% power at the 95% level of confidence to confirm/refute a 1.80‐fold increased odds of dementia associated with moderate/severe calcification (defined in the previous studies as >median level), assuming a prevalence rate among controls of 0.48 (0.47 and 0.49 in previous studies). A sample of 200 dementia cases is also consistent with a mean of 193 cases in the previous studies.^13^, ^16^

We therefore selected 200 cases with dementia during follow‐up after a TIA or minor stroke and 200 age‐ and sex‐matched controls without dementia on follow‐up. All cases and controls were selected from OXVASC participants recruited between April 1, 2002 and March 31, 2012 who had only a TIA or a minor stroke (National Institutes of Health Stroke Scale score <3).^3^ Cases were selected on the basis of having no premorbid dementia, no dementia on initial 1‐month follow‐up after the event, but who had a neurologist or psychiatrist confirmed diagnosis of dementia during follow‐up to 10 years thereafter. No subtype of dementia was specified. For each case, 1 control recruited during the same study year and imaged using the same CT scanner was selected manually using the following criteria: same sex, no dementia at the follow‐up time at which the case had developed dementia, and closest age to the case (±5 years) of those potential matches that satisfied the first 2 criteria. Time to death was similar in the cases and controls (log‐rank χ^2^=0.045, *P*=0.83).

### Calcification Measurements

Eligible participants had head CT scans on General Electric LightSpeed, Canon Aquilion, and Siemens Somatom machines at 120 to 140 kV and 50 to 381 mA, with most scans having section width of 2.5 to 4 mm for posterior fossa and 7.5 to 8 mm for supratentorial regions.

CT scans were viewed on an InSight PACS workstation (Intelerad Medical Systems, Montreal, Canada) or on hard‐copy films when electronic copies are not available. The internal carotid artery (ICA) was evaluated from the petrous apex to the terminal bifurcation, while the vertebral artery (VA) was assessed from the posterior atlantooccipital membrane to where bilateral VAs merge. Calcification was assessed using both qualitative visual scales and a semiautomated quantitative image analysis software. For visual scoring, window level/width were set at 40/80 and 400/800 Hounsfield units or similar values. Assessments were made blind to clinical and other radiological data.

Visual scoring of intracranial ICA calcification was based on the Woodcock scale,^33^ in which calcification is categorized into 4 ordinal grades: absent, mild (thin, discontinuous calcification), moderate (thin continuous or thick discontinuous calcification), and severe (thick, continuous calcification), corresponding to scores of 0, 1, 2, and 3, respectively. We also used the Woodcock scale to assess intracranial VA calcification. In the absence of definitions of thick/thin and continuous/discontinuous in the original report of the Woodcock scale,^33^ we defined thick calcification if it exceeded the width of the adjacent arterial wall. We defined continuous calcification if it was near‐circumferential (≥270°) on transverse sections, or tram‐track pattern on longitudinal sections, and was present in ≥50% of the arterial segment being assessed (Figures S1 through S5). CT‐IAC in the basilar artery (BA) was assessed using a binary (present/absent) classification, in line with previous literature.^34^ For the ICA and the VA, CT‐IAC of the right and left sides were scored separately, and a general score was derived as the highest score from either side. This approach was chosen over summing bilateral scores due to the nonlinear relationship between visual grades and actual calcification volume.^35^ Moreover, we determined the location subtype (dominant intimal versus dominant nonintimal) of ICA CT‐IAC using a visual scale that has been validated against histology.^36^ This scale was implemented on electronic CT images with window level/width set at 300/1600 Hounsfield units to classify ICA CT‐IAC based on its visual features including circularity, thickness, and morphology (Figure S6).^36^ Similar to a previous report from the Rotterdam Study,^13^ we recorded the CT‐IAC subtypes of the right and left ICA separately and divided patients into the atherosclerotic subtype (having only the intimal subtype of CT‐IAC in either or both sides of ICA), the IEL subtype (having only the nonintimal subtype of CT‐IAC in either or both sides of ICA), and the mixed subtype (having both the intimal and nonintimal subtypes of CT‐IAC).

Two raters participated independently in assessing calcification using the Woodcock and binary scales. The first rater (K.L.) had 4 years of neurology and neuroradiology training, and the second rater (D.S.) was a consultant neuroradiologist with 9 years of experience in radiology. K.L. assessed all CT scans, and D.S. also assessed equivocal cases. A randomly selected subset of scans was used to evaluate intra‐ and interrater reliability of the visual calcification scales, with KL reassessing 70 scans 3 months after his initial assessment, and both observers independently assessing 30 scans.

For comparability with previous studies^14^, ^15^, ^37^ semiautomated quantitative assessment of calcification was done in all participants in which electronic CT scan files were routinely available on an imaging workstation. For every participant, a region of interest of a structure was drawn in each slice with a threshold of ≥130 Hounsfield units for ICA, VA, and BA.^14^, ^15^, ^37^ CT‐IAC volume (in cubic millimeters) in a structure was calculated by multiplying the number of pixels above the corresponding threshold, size of a pixel, and slice increment. This was realized by the ‘Segment Editor’ and ‘Segment Statistics’ functions of 3D Slicer 5.6.2 (https://www.slicer.org/), with both itself and these functions validated.^38^, ^39^ Visual CT‐IAC measurements were correlated with the quantitative measures in 200 randomly selected participants. The association was used to impute quantitative measurements in those cases in whom electronic files were not available. The median CT‐IAC volume of each visual score was calculated with the right and left sides considered separately (Table S1) and was used to impute the calcification volume based on visual scores.

### Statistical Analysis

Student's *t* test was used to compare 2 independent samples when both followed a normal distribution; otherwise, the Mann–Whitney *U* test was applied. For proportion comparisons, the chi‐square test was used.

Logistic regression was used to assess the association of CT‐IAC with risk of dementia, and with cardiovascular and cerebrovascular events. Models were adjusted for age and sex only, or for age, sex, smoking, higher alcohol intake (defined as more than 14 units per week or history of alcohol abuse), medical histories (hypertension, diabetes, hyperlipidemia, atrial fibrillation, TIA, stroke, angina, myocardial infarction, and peripheral vessel disease), stroke severity (National Institutes of Health Stroke Scale), and the interval from the index event to CT scan (multivariable model). White matter lesion of presumed vascular origin (moderate and above versus others) was further adjusted to test the robustness of the results. The regression models for the main analysis were also rerun using conditional logistic regression to respect the matching structure.

Regression was repeated for participants without recurrent stroke during follow‐up as sensitivity analysis and repeated separately for participants who developed dementia during or beyond 5‐year follow‐up. Association between CT‐IAC and vascular events during follow‐up including unstable angina, myocardial infarction, sudden cardiac death, TIA, and stroke was also analyzed in the same manner to assess potential confounding and competing risks, as was the association between the IEL subtype of CT‐IAC and dementia further adjusted for the severity of CT‐IAC. Potential nonlinearity of the association between CT‐IAC volume and dementia risk was assessed using the restricted cubic splines with 4 knots based on Harrell's recommended percentiles^40^ and was realized by the “rms” package in R 4.3.2 (https://www.r‐project.org). Nagelkerke *R* ^2^ was used to assess goodness of fit of the linear and nonlinear models. Radiological reports from routine practice of the National Health Service, UK, in patients with severe CT‐IAC were reviewed to record the report rate of calcification in these patients.

Square‐weighted Cohen's κ was used to assess the intra‐ and interrater reliability of visual scales. Spearman's ρ was used to evaluate the correlation between visual calcification scores and quantitative calcification volume. Analyses were conducted using SPSS 29 (IBM, NY, USA) or R 4.3.2, with the significance level (α) set at 0.05 (2 tailed).

## RESULTS

Among the 400 participants with TIA/minor stroke in the longitudinal matched case–control study (Table 1), median age at index event was 78.5 (interquartile range, 71.8–84.5) years and the median interval between seeking medical attention and brain imaging was 1 day (interquartile range, 0–2 days). Participants who developed dementia on follow‐up (cases) and controls were matched as described in the Methods section, and the groups did not differ significantly in age, other demographics, or vascular risk factors. Median follow‐up time was 7.9 (interquartile range, 3.5–13.1) years.

**Table 1:** Baseline Characteristics of Participants Who Did and Did Not Develop Dementia During Follow‐Up

Item	Participants who developed dementia	Participants who did not develop dementia	*P* value
Sample size	200	200	/
Age, y	80.0 (73.0–84.7)	77.3 (71.1–84.0)	0.71
Female sex	107 (53.5%)	107 (53.5%)	1.00
Current smoker	20 (10.0%)	24 (12.0%)	0.62
Higher alcohol intake	27 (13.5%)	31 (15.5%)	0.67
Past medical history			
Hypertension	132 (66.0%)	130 (65.0%)	0.91
Hyperlipidemia	66 (33.0%)	63 (31.5%)	0.83
Diabetes	32 (16.0%)	27 (13.5%)	0.59
Atrial fibrillation	32 (16.0%)	29 (14.5%)	0.78
Transient ischemic attack	26 (13.0%)	30 (15.0%)	0.67
Stroke	22 (11.0%)	24 (12.0%)	0.75
Angina	36 (18.0%)	39 (19.5%)	0.80
Myocardial infarction	28 (14.0%)	25 (12.5%)	0.76
Peripheral vascular disease	18 (9.0%)	21 (10.5%)	0.71
National Institutes of Health Stroke Scale score	0 (0–1)	0 (0–1)	0.97
Moderate or severe white matter lesion	77 (38.5%)	58 (29.0%)	0.06

Values are counts (percentage) for categorical variables, and median (interquartile range) for age and National Institutes of Health Stroke Scale score. Higher alcohol intake is defined as >14 units per week or history of alcohol abuse. Paired comparisons between cases and controls were performed using McNemar's test for binary variables and paired *t* test or Wilcoxon signed‐rank test for continuous/ordinal variables.

The intra−/interrater reliability for assessment of calcification using the visual rating scale was good (Table S2), with Cohen's *κ* >0.80 for ICA and VA and ≥0.65 for BA. The correlation between calcification measures on the visual rating scale and semiautomated volume of CT‐IAC was also strong (ρ≥0.65) for ICA, VA, and BA (Table S2).

On visual scales (Figures S7 and S8), general scores for ICA were as follows: 0 in 10 individuals (2.5%), 1 in 35 individuals (8.8%), 2 in 174 individuals (43.5%), and 3 in 181 individuals (45.3%). For VA, 198 individuals (49.5%) scored 0, 80 (20.0%) scored 1, 95 (23.8%) scored 2, and 27 (6.8%) scored 3. Calcification of BA was present in 22 individuals (5.5%). On semiautomated quantitative measurement, median calcification volume was 49.0 (interquartile range, 35.4–421.4) mm^3^ for ICA and 0 (0–8.4) mm^3^ for vertebrobasilar arteries (VBA).

On the visual scale, bilateral severe ICA calcification was found in 73 (36.5%) participants who developed dementia and in 49 (24.5%) who did not, with a significant association with dementia risk on multivariable logistic regression (Table 2; adjusted odds ratio [aOR], 2.02 [95% CI, 1.26–3.23], *P*=0.004) Only 6 participants had bilateral severe VA calcification, and so we modeled the combined measure of calcification in the ICA and VBA (Table 2; aOR, 1.95 [95% CI, 1.22–3.12], *P*=0.005).

**Table 2:** Univariable and Multivariable Logistic Regression Analysis of Severity of Intracranial Arterial Calcification (Qualitative Visual Scale or Semiautomated Software) and Risk of Dementia

Calcification measures	Artery	Odds ratio for dementia (95% CI)							
		Univariable	*P* value	Age and sex adjusted	*P* value	Multivariable Model	*P* value	Multivariable model, WML adjusted	*P* value
Bilateral severe calcification (qualitative)	ICA	1.77 (1.15–2.73)*	0.010*	1.85 (1.18–2.89)*	0.007*	2.02 (1.26–3.23)*	0.004*	1.98 (1.23–3.18)*	0.005*
	ICA or vertebral artery	1.72 (1.12–2.65)*	0.013*	1.80 (1.15–2.80)*	0.010*	1.95 (1.22–3.12)*	0.005*	1.92 (1.20–3.07)*	0.007*
Top vs bottom tertile calcification volume (quantitative)	ICA	1.99 (1.22–3.26)*	0.006*	2.05 (1.23–3.40)*	0.006*	2.35 (1.33–4.16)*	0.003*	2.34 (1.33–4.14)*	0.003*
	VBA	1.77 (0.69–4.54)	0.24	1.74 (0.66–4.55)	0.26	2.29 (0.58–9.06)	0.24	2.24 (0.56–8.93)	0.25
	ICA and VBA	2.13 (1.28–3.54)*	0.004*	2.12 (1.26–3.59)*	0.005*	2.59 (1.43–4.68)*	0.002*	2.57 (1.42–4.67)*	0.002*

Bilateral severe calcification means the highest score in both sides on the qualitative visual scale. Top and bottom tertiles in volume are based on sem‐automated calcification volume in participants with prevalent calcification. The multivariable models adjusted for age, sex, smoking, higher alcohol intake, medical histories (hypertension, diabetes, hyperlipidemia, transient ischemic attack, stroke, angina, myocardial infarction, atrial fibrillation, and peripheral vessel disease), National Institutes of Health Stroke Scale score, and the interval from index stroke or transient ischemic attack to computed tomography scan. Higher alcohol intake is defined as >14 units per week or history of alcohol abuse. WML (moderate and above vs others) was further adjusted to test the robustness of the results. ICA indicates internal carotid artery; VBA, vertebrobasilar artery; and WML, white matter lesion.

* *P* < 0.05.

The semiautomated quantitative measurement of CT‐IAC was also independently associated with dementia for ICA calcification (top versus bottom tertile—aOR, 2.35 [95% CI, 1.33–4.16], *P*=0.003), VBA calcification (aOR, 2.29 [95% CI, 0.58–9.06], *P*=0.24), and total CT‐IAC (aOR, 2.59 [95% CI, 1.43–4.68], *P*=0.002). Similar results were seen after adjusting for white matter lesion (Table 2) or excluding patients with moderate or severe white matter disease (top versus bottom tertile total CT‐IAC—aOR, 3.19 [95% CI, 1.54–6.62], *P*=0.002). There was no significant association between CT‐IAC and risk of recurrent stroke during follow‐up (Table S3), and excluding participants who had stroke during follow‐up (n=83) did not change the associations between CT‐IAC and dementia (Table S4). CT‐IAC measures were also not significantly related to either a previous history of coronary events or to the risk of acute coronary events during follow‐up (Table S3). The associations remained unchanged for patients who developed dementia before versus after 5‐year follow‐up (Table S5). Repeating the main analyses using conditional logistic regression, given the matching on age and sex, also yielded similar results. (Table S6).

**Figure 1:**
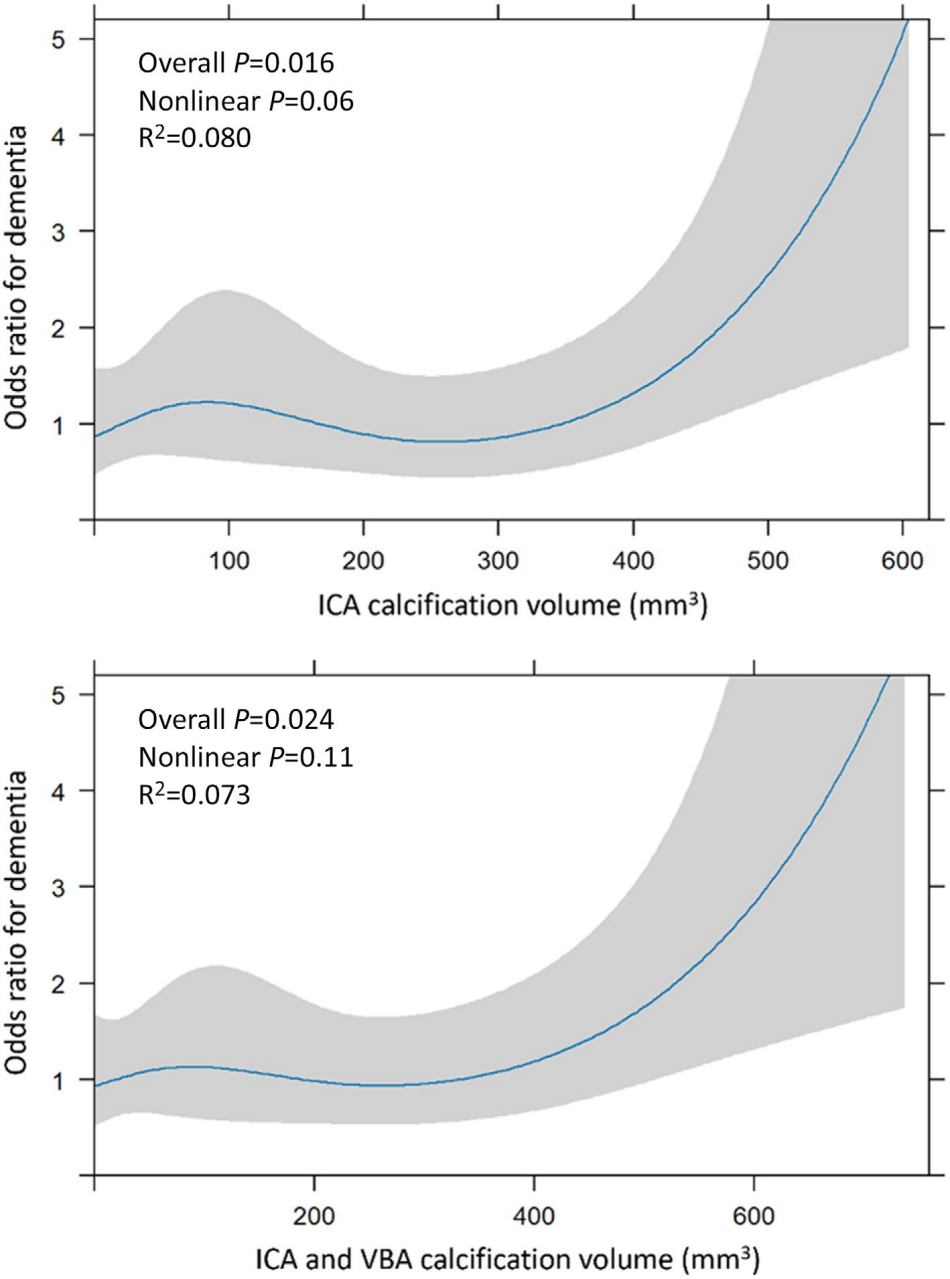
Restricted cubic spline models for intracranial arterial calcification and risk of dementia. Models were adjusted for age, sex, smoking, higher alcohol intake, medical histories (hypertension, diabetes, hyperlipidemia, transient ischemic attack, stroke, angina, myocardial infarction, atrial fibrillation, and peripheral vessel disease), National Institutes of Health Stroke Scale score, and the interval from index stroke or transient ischemic attack to computed tomography scan. Higher alcohol intake is defined as >14 units per week or history of alcohol abuse. Nagelkerke *R* ^2^ was used to assess goodness of fit of the linear and nonlinear models. ICA indicates internal carotid artery; and VBA, vertebrobasilar artery.

The association between CT‐IAC volume and dementia appeared to be nonlinear, with much of the association driven by the small subgroup with very high calcification volumes (Table 3; ≥600 mm^3^ versus 0–299 mm^3^—aOR, 6.23 [95% CI, 1.24–31.24], *P*=0.026), although it did not differ statistically from a linear association (ICA calcification volume—nonlinear *P*=0.06; total CT‐IAC—nonlinear *P*=0.11) on the restricted cubic spline model. Nevertheless, the restricted cubic spline model did result in a somewhat better fit than a linear model for both ICA calcification volume (R^2^=0.080 versus 0.058) and for total CT‐IAC (R^2^=0.073 versus 0.057) (Figure1).

**Table 3:** Univariable and Multivariable Logistic Regression Analysis of Categories of Intracranial Arterial Calcification Volume and Risk of Dementia

Calcification measures	Participant numbers	Categories	Odds ratio for dementia (95% CI)		
			Univariable	Age and sex adjusted	Multivariable Model
ICA calcification volume	277	0–299 mm^3^	Reference	Reference	Reference
	112	300–599 mm^3^	1.50 (0.96–2.33)	1.54 (0.98–2.42)	1.70 (1.05–2.76)*
	11	≥600 mm^3^	5.24 (1.11–24.69)*	5.52 (1.16–26.28)*	6.23 (1.24–31.24)*
VBA calcification volume	369	0–49 mm^3^	Reference	Reference	Reference
	21	50–199 mm^3^	1.39 (0.57–3.37)	1.39 (0.57–3.38)	1.58 (0.62–3.99)
	10	≥200 mm^3^	2.42 (0.62–9.52)	2.44 (0.62–9.58)	2.18 (0.53–8.88)
ICA and VBA calcification volume	269	0–299 mm^3^	Reference	Reference	Reference
	109	300–599 mm^3^	1.38 (0.88–2.16)	1.42 (0.90–2.24)	1.57 (0.97–2.54)
	22	≥600 mm^3^	3.98 (1.43–11.09)*	4.14 (1.47–11.63)*	4.56 (1.57–13.26)*

The multivariable models adjusted for age, sex, smoking, higher alcohol intake, medical histories (hypertension, diabetes, hyperlipidemia, transient ischemic attack, stroke, angina, myocardial infarction, atrial fibrillation, and peripheral vessel disease), National Institutes of Health Stroke Scale score, and the interval from index stroke or transient ischemic attack to computed tomography scan. Higher alcohol intake is defined as >14 units per week or history of alcohol abuse. ICA indicates internal carotid artery; and VBA, vertebrobasilar artery.

* *P* < 0.05.

Regarding the location subtype of CT‐IAC in ICA on visual assessment, CT‐IAC of the IEL‐only type was present in 78 (39.0%) of the dementia group versus 55 (27.5%) for the control group whereas the intimal‐only type was seen in 77 (38.5%) of the dementia group versus 93 (46.5%) in the control group. The IEL location of ICA CT‐IAC itself predicted dementia independent of vascular risk factors and the overall ICA calcification severity (qualitative severity adjusted—aOR=1.84, 1.11–3.05, *P*=0.019; log‐transformed calcification volume adjusted—aOR=1.78, 1.06–2.99, *P*=0.029).

On going through the routine radiological reports by National Health Service neuroradiologists, we found that of the 185 patients with severe CT‐IAC in either side of the intracranial ICA or VA based on the visual scales, CT‐IAC was mentioned in the routine report in only 6 (3.2%) instances.

## DISCUSSION

Two previous longitudinal studies showed that calcification in ICA/VBA tended to increase dementia risk during follow‐up, 1 in patients with stroke/TIA and 1 in the general population, but most effect sizes were of borderline statistical significance.^13^, ^16^ The longitudinal matched case–control analysis nested in OXVASC has validated these 2 previous reports by also showing that CT‐IAC severity in the ICA significantly increased dementia risk during follow‐up.

Although the association between CT‐IAC and dementia appears to be independent of vascular risk factors, a causal link cannot be assumed. However, there is some evidence of plausible mechanisms. For example, in mouse models, arterial stiffness due to carotid calcification led to compromised resting cerebral blood flow and cerebral autoregulation, and increased pulsatility, blood–brain barrier permeability, and Aβ40/Aβ42 ratio.^41^, ^42^ A recent report from the Rotterdam Study showed a somewhat stronger association with dementia for CT‐IAC of the medial/IEL type versus CT‐IAC of the atherosclerotic intimal type, potentially supporting mediation by arterial stiffness rather than hypoperfusion and microembolism,^13^ and our current study also showed that IEL calcification in ICA independently predicted dementia on follow‐up. Indeed, although CT‐IAC is probably also an independent predictor of ischemic stroke,^9^ the associations with dementia in the Rotterdam Study and in OXVASC were independent of stroke recurrence. The finding that calcification of the aortic arch and of the extracranial or intracranial ICA appear to be more strongly related to future cognitive decline and dementia than coronary artery calcification^14^, ^25^ would also be consistent with greater predictive value of medial/IEL versus atherosclerotic intimal calcification.

In OXVASC, the association between CT‐IAC and dementia risk appeared to be nonlinear, with risk concentrated in the 2% to 3% of individuals with very high absolute calcification volumes. The association reported in the Rotterdam Study was based on log‐transformed absolute CT‐IAC volume, which might also suggest similar nonlinearity. More research is required to determine the shape of the association both with risk of dementia and also with potential mediators, such as arterial stiffness and pulsitility. However, the fact that only severe calcification appears to substantially increase the risk of dementia means that simple visual scales should be adequate to identify at risk individuals in routine practice. The Woodcock scale has been widely used and validated,^33^, ^35^ and had good intra−/interrater κ for ICA and VA calcification in OXVASC.

Although our study has some strengths, including the population‐based sampling, good reproducibility of the assessment of CT‐IAC, and use of both visual and semiautomated quantitative measures, there are some limitations. First, in common with previous studies, we were not able to fully account for confounding by medications, such as loop diuretics,^43^, ^44^ warfarin,^45^ and angiotensin receptor blockers,^46^, ^47^ which are potentially related to both vascular calcification and dementia. Second, the OXVASC cohort is predominantly White, as is the Rotterdam cohort, and so the findings might not be fully generalizable. Third, in some patients recruited in the early phase of OXVASC, only hard copies of CT images were available and so only visual scales of CT‐IAC could be used. However, the visual scales had good intra−/interrater reliability and were strongly correlated with semiautomated CT‐IAC volume in those patients who had both measures. Moreover, visual assessment might be easier to implement in routine clinical practice.^35^ Finally, the association of dementia with particularly high levels of calcification raises the question of confounding by metabolic factors, such as renal dysfunction, vitamin D deficiency, or parathyroid dysfunction, which were not adjusted for in OXVASC or in previous longitudinal studies. Future studies should ideally address potential metabolic confounders.

## CONCLUSIONS

Our findings strengthen the evidence base on the positive association between the severity of ICA/VBA calcification and future risk of dementia in patients with stroke or TIA, independent of baseline stroke severity/recurrence and severity of white matter lesions. However, our review of the prior routine reports of the scans used in OXVASC showed that severe CT‐IAC was generally not mentioned. Larger studies are needed to further elucidate the apparent nonlinearity of the association, underlying mechanisms, and any therapeutic implications, but routine reporting of severe CT‐IAC in patients with stroke/TIA could be justified in the meantime.

## Sources of Funding

Dr Ke (Michael) Li is supported by the Clarendon Fund, Balliol College, and the China Oxford Scholarship Fund. The Oxford Vascular Study is supported by grants to Professor Rothwell from the Wellcome Trust and the National Institute for Health and Care Research Oxford Biomedical Research Centre.

## Disclosures

None.

## Supporting information

Tables S1–S6
Figures S1–S8

STROBE Checklist
