# Supplementary material for: Severity of Intracranial Arterial Calcification on Computed Tomography and Risk of Dementia in Patients With Stroke or Transient Ischemic Attack: A Population‐Based Study
Source: J Am Heart Assoc. 2026 Mar 4;15(6):e046801. doi: 10.1161/JAHA.125.046801 (PMC13055789; doi:10.1161/JAHA.125.046801)

Supplemental Material

Table S1. Median calcification volume by score on qualitative scales for imputation.

		Median calcification volume (mm^3) by score			
**Structure**	**Details**	**0**	**1**	**2**	**3**
ICA	Right	0.00	0.00	19.61	206.49
	Left	0.00	0.00	18.62	214.89
VA	Right	0.00	0.00	11.29	140.33
	Left	0.00	0.00	8.35	140.53
BA	130 HU	0.00	0.00	/	/
	90 HU	0.00	1.59	/	/

Median ICA, VA, and BA calcification volume were calculated for each score on the qualitative scales in 200 random participants from the Oxford Vascular Study. BA, basilar artery; HU, Hounsfield unit; ICA, internal carotid artery; IQR, interquartile range; VA, vertebral artery.

Table S2. Reliability of qualitative calcification scales and correlation between qualitative scales and semi-automated calcification volume.

Structure	Parameter	Category	Point estimation (95% confidence interval)	p-value
ICA	Cohen's κ	Intra-rater	0.83 (0.67-0.99)	<0.001
		Inter-rater	0.83 (0.58-1.00)	<0.001
	Percent agreement	Intra-rater	80.0%	/
		Inter-rater	76.7%	/
	Spearman's ρ	Right score	0.78	<0.001
		Left score	0.79	<0.001
		General score	0.79	<0.001
VA	Cohen's κ	Intra-rater	0.83 (0.67-1.00)	<0.001
		Inter-rater	0.85 (0.60-1.00)	<0.001
	Percent agreement	Intra-rater	82.1%	/
		Inter-rater	76.7%	/
	Spearman's ρ	Right score	0.71	<0.001
		Left score	0.70	<0.001
		General score	0.79	<0.001
BA	Cohen's κ	Intra-rater	0.79 (0.56-1.00)	<0.001
		Inter-rater	0.65 (0.31-0.99)	<0.001
	Percent agreement	Intra-rater	97.1%	/
		Inter-rater	96.7%	/
	Spearman's ρ	Binary score	0.65	<0.001

According to the OXVASC study number, 70 and 30 randomly selected participants recruited during 1 April 2002 and 31 March 2012 were randomly selected for evaluating intra- and inter-rater reliability, respectively. For correlation of qualitative and quantitative calcification measurements using Spearman's ρ, 200 randomly selected participants from OXVASC recruited in the same period were included. On semi-automated software, threshold for calcification was ≥130 HU. The general score equals to the highest score of either side for bilateral structures. BA, basilar artery; ICA, intracranial artery; OXVASC, Oxford Vascular Study; VA, vertebral artery.

Table S3. Univariable and multivariable logistic regression analysis of severity of intracranial arterial calcification (qualitative visual scale or semi-automated software) and vascular events at baseline and on follow-up.

			Odds ratio for dementia (95% confidence interval)					
**Calcification measures**	**Artery**	**Cardiac outcomes**	**Univariable model**	**p-value**	**Age and sex adjusted**	**p-value**	**Multivariable model**	**p-value**
Bilateral severe calcification	ICA or VA	History of MI	1.73 (0.96-3.13)	0.07	1.74 (0.94-3.22)	0.08	1.50 (0.75-2.97)	0.25
		History of stroke	1.23 (0.64-2.35)	0.53	1.36 (0.69-2.67)	0.37	1.31 (0.64-2.72)	0.46
		UA/MI/SCD on follow-up	1.27 (0.76-2.13)	0.35	1.26 (0.74-2.14)	0.40	1.20 (0.68-2.12)	0.53
		TIA/stroke on follow-up	1.33 (0.87-2.04)	0.19	1.50 (0.97-2.34)	0.07	1.43 (0.90-2.28)	0.13
Top vs. bottom tertile calcification volume	ICA and VBA	History of MI	1.93 (0.94-3.97)	0.07	2.05 (0.97-4.35)	0.06	1.81 (0.76-4.28)	0.18
		History of stroke	1.49 (0.67-3.29)	0.33	1.75 (0.77-3.99)	0.18	1.99 (0.81-4.93)	0.14
		UA/MI/SCD on follow-up	1.09 (0.61-1.95)	0.77	1.09 (0.59-1.99)	0.78	1.06 (0.54-2.08)	0.86
		TIA/stroke on follow-up	1.47 (0.88-2.43)	0.14	1.66 (0.98-2.82)	0.06	1.58 (0.89-2.79)	0.12

Bilateral severe calcification means the highest score in both sides on the qualitative visual scale. Top and bottom tertiles in volume are based on semi-automated calcification volume in participants with prevalent calcification. The multivariable models adjusted for age, sex, smoking, higher alcohol intake, medical histories (hypertension, diabetes, hyperlipidaemia, TIA/stroke [for cerebrovascular outcomes, they were not adjusted], angina/MI [for cardiac outcomes, they were not adjusted], atrial fibrillation, and peripheral vessel disease), National Institutes of Health Stroke Score, and the interval from index stroke or transient ischaemic attack to computed tomography scan. Higher alcohol intake is defined as more than 14 units per week or history of alcohol abuse. ICA, internal carotid artery; MI, myocardial infarction; SCD, sudden cardiac death; TIA, transient ischaemic attack; UA, unstable angina; VA, vertebral artery; VBA, vertebrobasilar artery.

Table S4. Univariable and multivariable logistic regression analysis of severity of intracranial arterial calcification (qualitative visual scale or semi-automated software) and risk of dementia with recurrent stroke censored.

**Calcification measures**		**Artery**	**Odds ratio for dementia (95% confidence interval)**					
			**Univariable model**	**p-value**	**Age and sex adjusted**	**p-value**	**Multivariable model**	**p-value**
Qualitative	Bilateral severe calcification	ICA	**1.77 (1.09-2.88)**	**0.021**	**1.89 (1.14-3.12)**	**0.013**	**2.05 (1.21-3.48)**	**0.008**
		ICA or VA	**1.71 (1.05-2.77)**	**0.030**	**1.82 (1.10-2.99)**	**0.019**	**1.96 (1.16-3.32)**	**0.012**
Quantitative	Top vs. bottom tertile calcification volume	ICA	**2.00 (1.14-3.49)**	**0.015**	**2.04 (1.15-3.64)**	**0.016**	**2.33 (1.22-4.44)**	**0.010**
		VBA	1.82 (0.63-5.30)	0.27	1.71 (0.57-5.14)	0.34	3.10 (0.41-23.41)	0.27
		ICA and VBA	**2.20 (1.23-3.92)**	**0.008**	**2.18 (1.20-3.95)**	**0.011**	**2.60 (1.33-5.08)**	**0.005**

Bilateral severe calcification means the highest score in both sides on the qualitative visual scale. Top and bottom tertiles in volume are based on semi-automated calcification volume in participants with prevalent calcification. Participants with recurrent stroke were excluded from the analysis. The multivariable models adjusted for age, sex, smoking, higher alcohol intake, medical histories (hypertension, diabetes, hyperlipidaemia, transient ischaemic attack, stroke, angina, myocardial infarction, atrial fibrillation, and peripheral vessel disease), National Institutes of Health Stroke Score, and the interval from index stroke or transient ischaemic attack to computed tomography scan. Higher alcohol intake is defined as more than 14 units per week or history of alcohol abuse. ICA, internal carotid artery; VA, vertebral artery; VBA, vertebrobasilar artery.

Table S5. Univariable and multivariable logistic regression analysis of severity of intracranial arterial calcification (qualitative visual scale or semi-automated software) and risk of dementia in patients who developed dementia during or beyond 5-year follow-up.

			Odds ratio for dementia (95% confidence interval)					
**Time of dementia diagnosis**	**Calcification measures**	**Artery**	**Age and sex adjusted**	**p-value**	**Multivariable Model**	**p-value**	**Multivariable Model, WML adjusted**	**p-value**
Within 5-year follow-up	Bilateral severe calcification (qualitative)	ICA	**1.95 (1.17-3.25)**	**0.010**	**2.16 (1.24-3.75)**	**0.006**	**2.17 (1.25-3.79)**	**0.006**
		ICA or VA	**1.91 (1.15-3.17)**	**0.012**	**2.11 (1.22-3.66)**	**0.008**	**2.13 (1.23-3.70)**	**0.007**
	Calcification volume >300 mm^3 (quantitative)	ICA	1.65 (0.99-2.75)	0.06	**1.75 (1.00-3.07)**	**0.048**	**1.80 (1.02-3.15)**	**0.041**
		ICA and VBA	1.57 (0.95-2.60)	0.08	1.66 (0.96-2.89)	0.07	1.71 (0.98-2.98)	0.06
Beyond 5-year follow-up	Bilateral severe calcification (qualitative)	ICA	1.79 (0.97-3.30)	0.06	**2.01 (1.04-3.88)**	**0.039**	1.92 (0.99-3.74)	0.05
		ICA or VA	1.72 (0.94-3.15)	0.08	1.88 (0.98-3.62)	0.06	1.81 (0.93-3.49)	0.08
	Calcification volume >300 mm^3 (quantitative)	ICA	1.81 (1.00-2.39)	0.05	**2.07 (1.08-3.97)**	**0.028**	**1.99 (1.03-3.83)**	**0.040**
		ICA and VBA	**1.89 (1.06-3.37)**	**0.032**	**2.10 (1.12-3.93)**	**0.020**	**2.05 (1.09-3.84)**	**0.025**

Bilateral severe calcification means the highest score in both sides on the qualitative visual scale. The multivariable models adjusted for age, sex, smoking, higher alcohol intake, medical histories (hypertension, diabetes, hyperlipidaemia, transient ischaemic attack, stroke, angina, myocardial infarction, atrial fibrillation, and peripheral vessel disease), National Institutes of Health Stroke Score, and the interval from index stroke or transient ischaemic attack to computed tomography scan. Higher alcohol intake is defined as more than 14 units per week or history of alcohol abuse. WML (moderate and above versus others) was further adjusted to test the robustness of the results. ICA, internal carotid artery; WML, white matter lesion; VA, vertebral artery; VBA, vertebrobasilar artery.

Table S6. Unconditional and conditional logistic regression analysis of severity of intracranial arterial calcification (qualitative visual scale or semi-automated software) and risk of dementia.

Logistic regression models	Calcification measures	Artery	Odds ratio for dementia (95% confidence interval)					
			**Age and sex adjusted/ matched**	**p-value**	**Multivariable Model**	**p-value**	**Multivariable Model, WML adjusted**	**p-value**
Unconditional	Bilateral severe calcification (qualitative)	ICA	**1.85 (1.18-2.89)**	**0.007**	**2.02 (1.26-3.23)**	**0.004**	**1.98 (1.23-3.18)**	**0.005**
		ICA or VA	**1.80 (1.15-2.80)**	**0.010**	**1.95 (1.22-3.12)**	**0.005**	**1.92 (1.20-3.07)**	**0.007**
	Calcification volume >300 mm^3 (quantitative)	ICA	**1.69 (1.09-2.63)**	**0.019**	**1.87 (1.17-3.00)**	**0.009**	**1.85 (1.15-2.98)**	**0.011**
		ICA and VBA	**1.66 (1.08-2.55)**	**0.021**	**1.83 (1.15-2.90)**	**0.011**	**1.83 (1.15-2.90)**	**0.011**
Conditional	Bilateral severe calcification (qualitative)	ICA	**1.86 (1.17-2.94)**	**0.008**	**1.95 (1.19-3.21)**	**0.009**	**1.89 (1.14-3.13)**	**0.013**
		ICA or VA	**1.79 (1.14-2.82)**	**0.012**	**1.82 (1.13-3.03)**	**0.014**	**1.81 (1.10-2.96)**	**0.019**
	Calcification volume >300 mm^3 (quantitative)	ICA	**1.72 (1.09-2.72)**	**0.020**	**1.77 (1.07-2.93)**	**0.025**	**1.72 (1.04-2.85)**	**0.035**
		ICA and VBA	**1.70 (1.08-2.67)**	**0.021**	**1.70 (1.04-2.78)**	**0.035**	**1.68 (1.02-2.76)**	**0.041**

Bilateral severe calcification means the highest score in both sides on the qualitative visual scale. Above and below median in volume are based on semi-automated calcification volume. The unconditional multivariable models adjusted for age, sex, smoking, higher alcohol intake, medical histories (hypertension, diabetes, hyperlipidaemia, transient ischaemic attack, stroke, angina, myocardial infarction, atrial fibrillation, and peripheral vessel disease), National Institutes of Health Stroke Score, and the interval from index stroke or transient ischaemic attack to computed tomography scan. Higher alcohol intake is defined as more than 14 units per week or history of alcohol abuse. WML (moderate and above versus others) was further adjusted to test the robustness of the results. The conditional models were matched for but not adjusted for age and sex, with other components similar to the unconditional models. ICA, internal carotid artery; WML, white matter lesion; VA, vertebral artery; VBA, vertebrobasilar artery.

Figure S1. Demonstration of patients with absent calcification (zero point) in intracranial ICA and VA on Woodcock Scale. (A) No calcification was seen in the left intracranial ICA (arrow). (B) No calcification was seen in the left intracranial VA (arrow). Window level and width were set at 400 HU and 800 HU, or 40 HU and 80 HU. HU, Hounsfield unit; ICA, internal carotid artery; VA, vertebral artery.

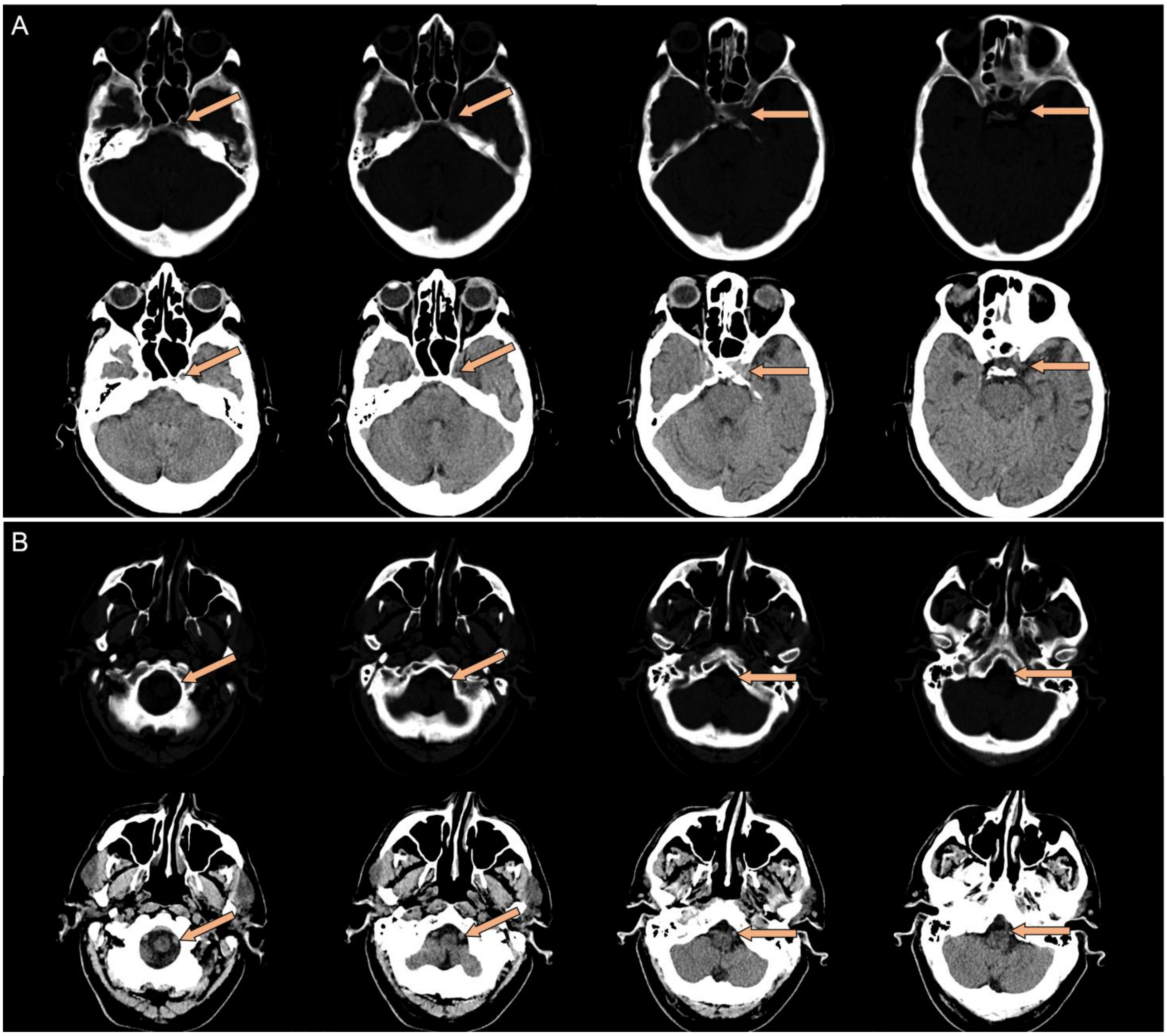

Figure S2. Demonstration of patients with thin discontinuous (mild) calcification (one point) in intracranial ICA and VA on Woodcock Scale. ‘Thin’ means the calcification does not exceed the width of the adjacent arterial wall. ‘Discontinuous’ means the calcification is not near-circumferential, tram-track-like, or present in ≥50% of the full length of the assessed vessel segments. (A) Thin and discontinuous calcification was seen in the right intracranial ICA (arrow). (B) Thin and discontinuous calcification was seen in the right intracranial VA (arrow). Window level and width were set at 400 HU and 800 HU, or 40 HU and 80 HU. HU, Hounsfield unit; ICA, internal carotid artery; VA, vertebral artery.

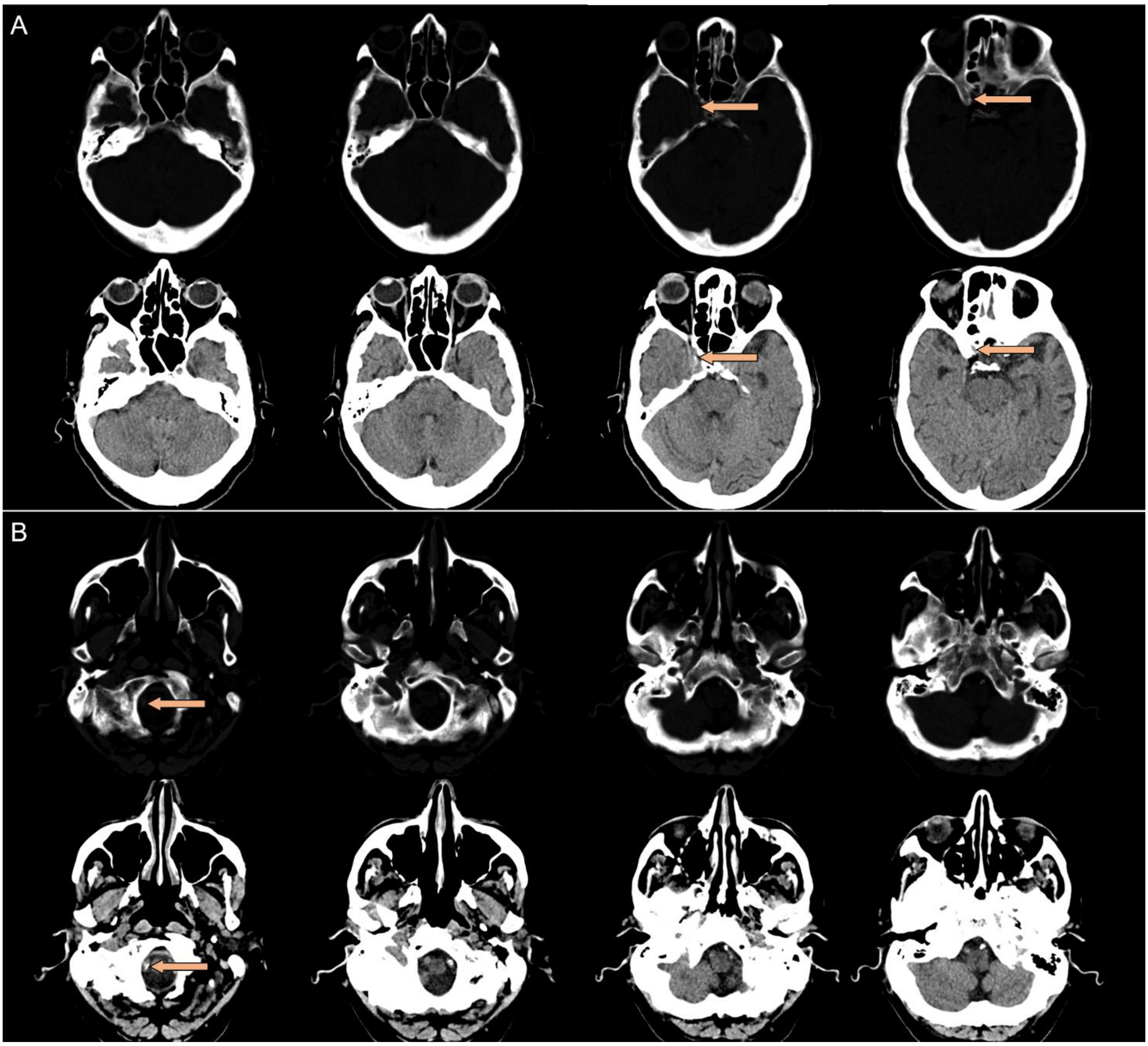

Figure S3. Demonstration of patients with thin continuous (moderate) calcification (two points) in intracranial ICA and VA on Woodcock Scale. 'Thin' means the calcification does not exceed the width of the adjacent arterial wall. 'Continuous' means the calcification is near-circumferential or tram-track-like, and is present in ≥50% of the full length of the assessed vessel segments. (A) Thin and continuous calcification was seen in the right intracranial ICA (arrow). (B) Thin and continuous calcification was seen in the right intracranial VA (arrow). Window level and width were set at 400 HU and 800 HU, or 40 HU and 80 HU. HU, Hounsfield unit; ICA, internal carotid artery; VA, vertebral artery.

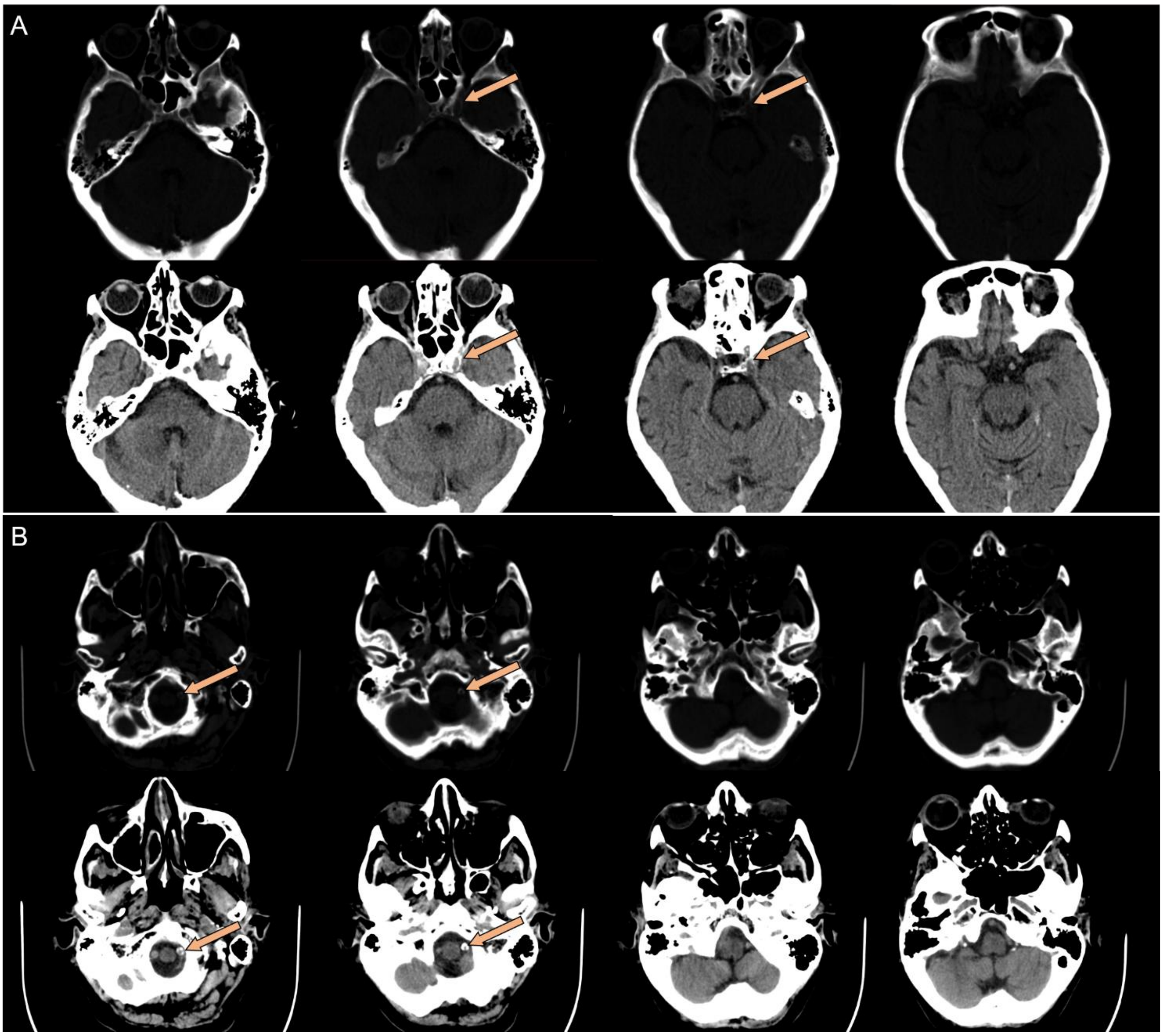

Figure S4. Demonstration of patients with thick discontinuous (moderate) calcification (two points) in intracranial ICA and VA on Woodcock Scale. ‘Thick’ means the calcification exceeds the width of the adjacent arterial wall. ‘Discontinuous’ means the calcification is not near-circumferential, tram-track-like, or present in ≥50% of the full length of the assessed vessel segments. (A) Thick and discontinuous calcification was seen in the right intracranial ICA (arrow). (B) Thick and discontinuous calcification was seen in the right intracranial VA (arrow). Window level and width were set at 400 HU and 800 HU, or 40 HU and 80 HU. HU, Hounsfield unit; ICA, internal carotid artery; VA, vertebral artery.

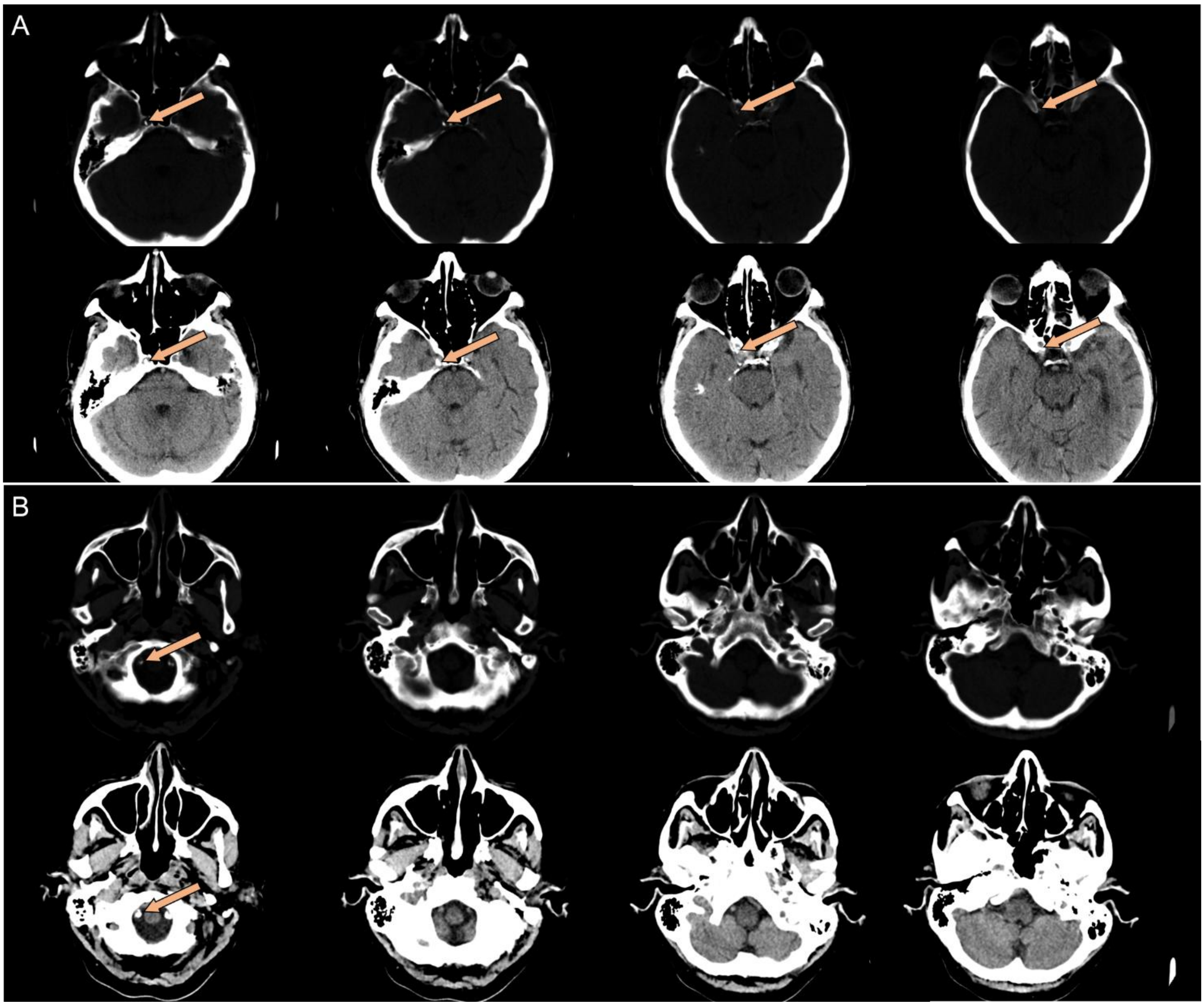

Figure S5. Demonstration of patients with thick continuous (severe) calcification (three points) in intracranial ICA and VA on Woodcock Scale. 'Thick' means the calcification exceeds the width of the adjacent arterial wall. 'Continuous' means the calcification is near-circumferential or tram-track-like, and is present in ≥50% of the full length of the assessed vessel segments. (A) Thick and continuous calcification was seen in the right intracranial ICA (arrow). (B) Thick and continuous calcification was seen in the right intracranial VA (arrow). Window level and width were set at 400 HU and 800 HU, or 40 HU and 80 HU. HU, Hounsfield unit; ICA, internal carotid artery; VA, vertebral artery.

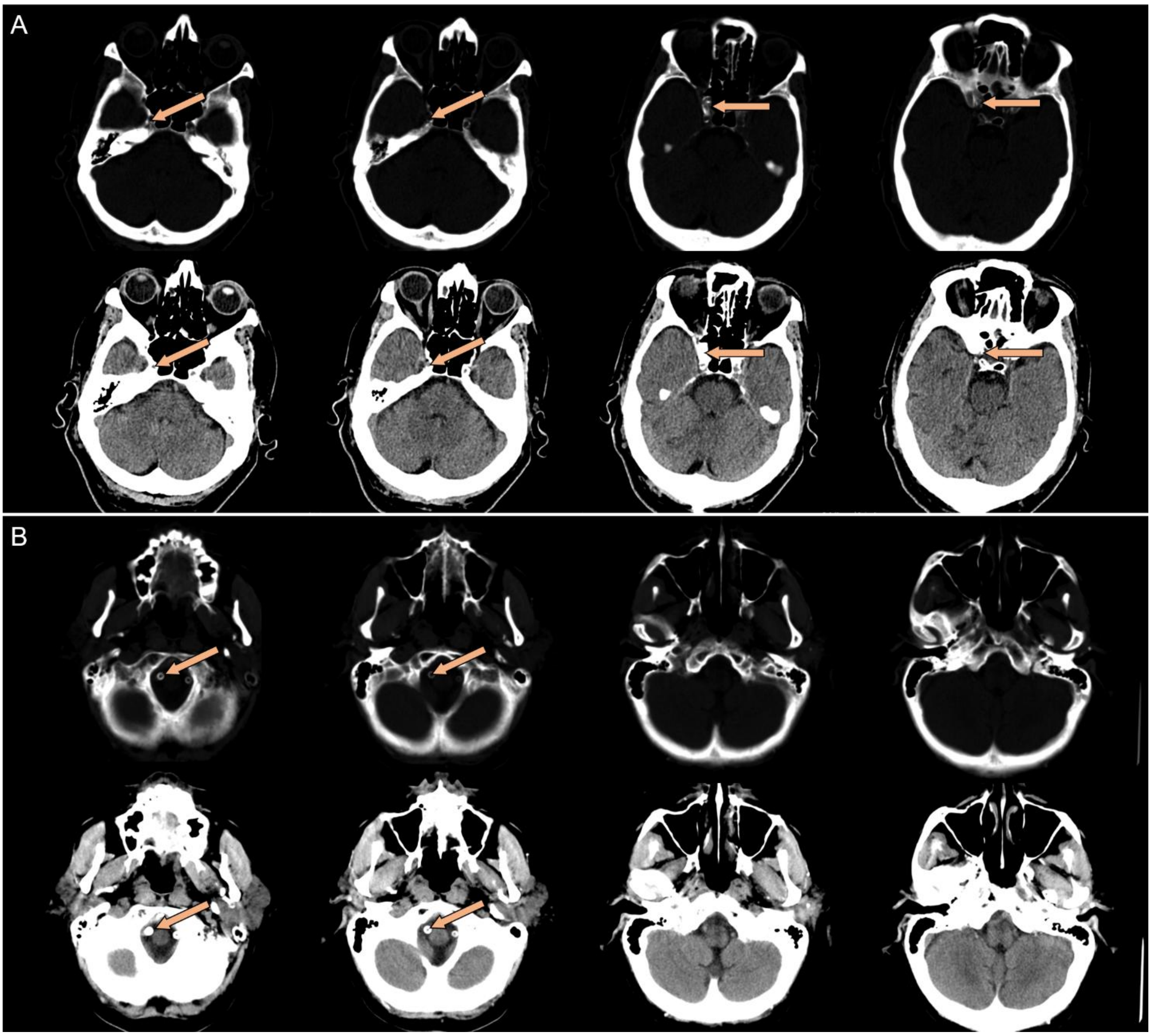

Figure S6. Demonstration of patients with (A) the dominant intimal subtype and (B) the dominant non-intimal subtype of calcification in both sides of their internal carotid arteries.

Calcification of the dominant intimal subtype tends to be non-circular, thick, and patchy, while that of the dominant non-intimal subtype is often circular, thin, and continuous. Window level and width were set at 300 HU and 1600 HU. HU, Hounsfield unit.

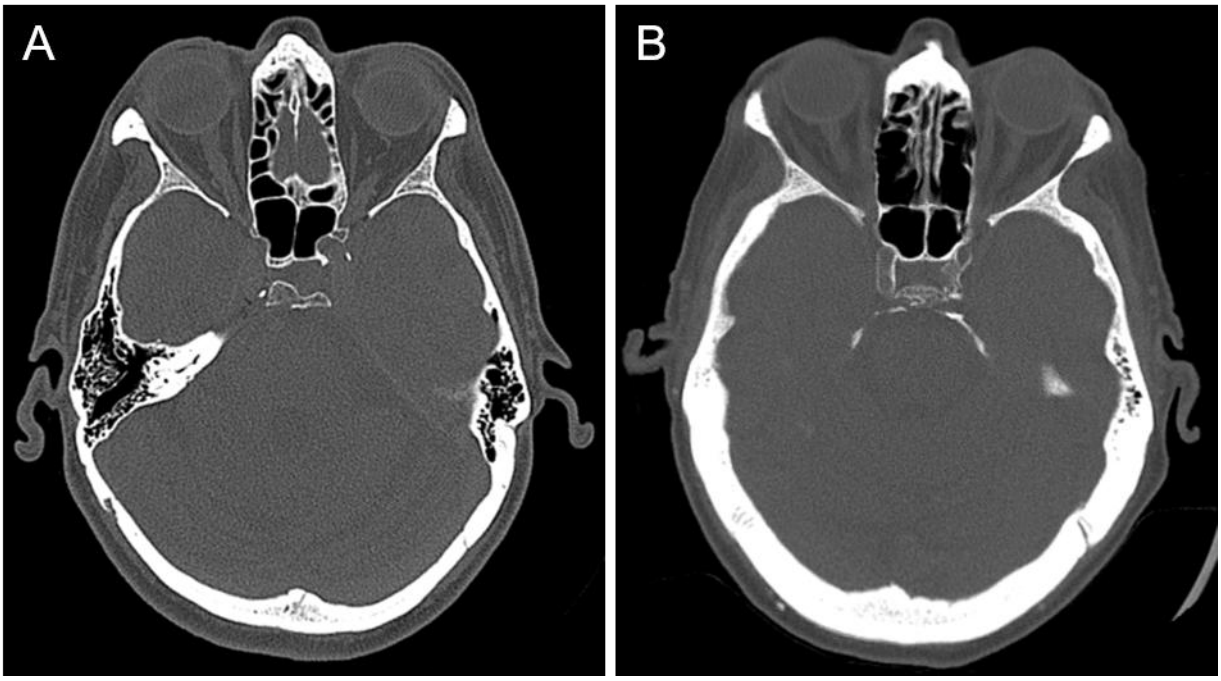

Figure S7. Calcification scores of ICA and VA assessed using the visual scales. The 'general' score equals the highest score from either side for bilateral structures. ICA, internal carotid artery; VA, vertebral artery.

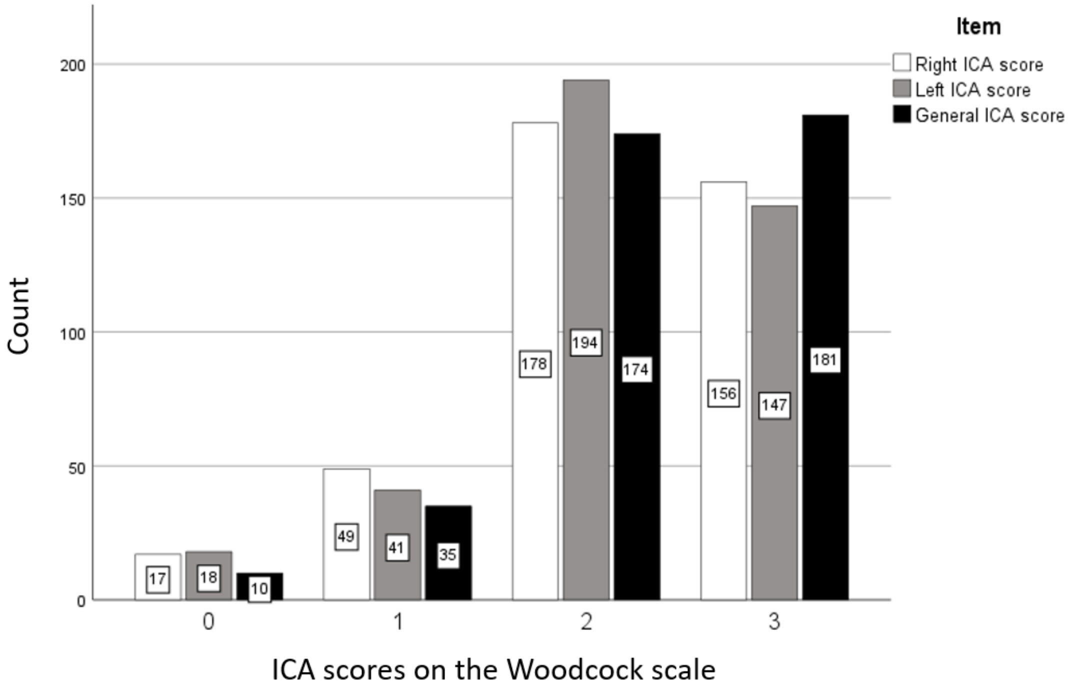

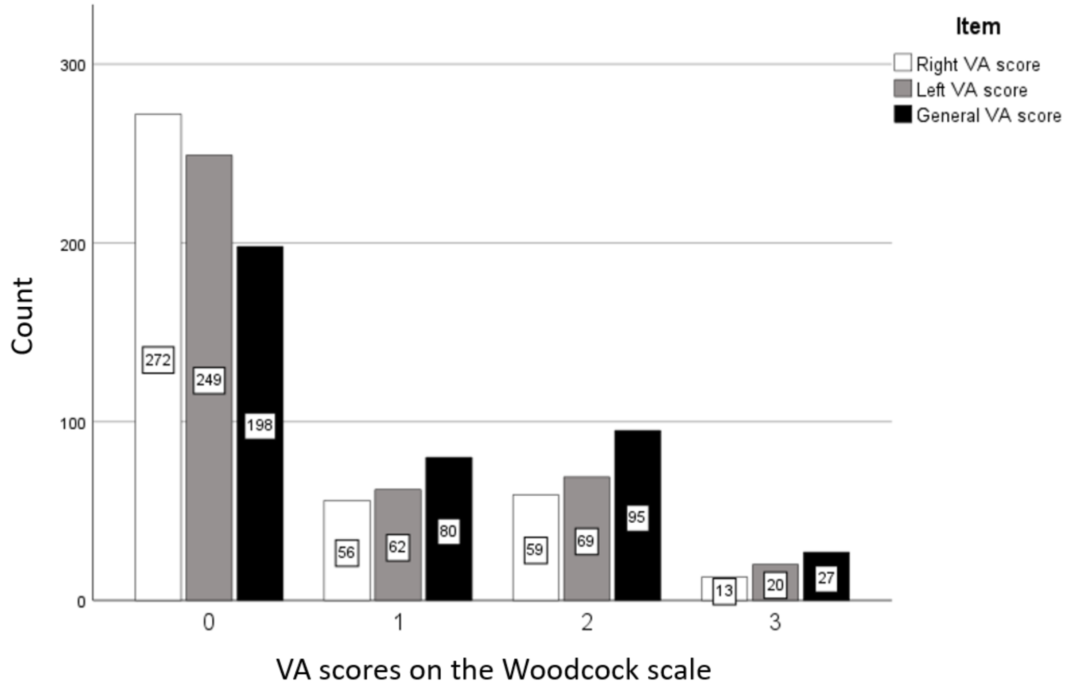

Figure S8. Calcification volume of ICA and VA assessed using the semi-automated software. In each patient, a region of interest of a structure was drawn in each slice with a threshold of ≥130 HU for ICA and VA. Calcification volume (in cubic millimetres) in a structure was calculated by multiplying the number of pixels above the corresponding threshold, size of a pixel, and slice increment. ICA, internal carotid artery; VA, vertebral artery.

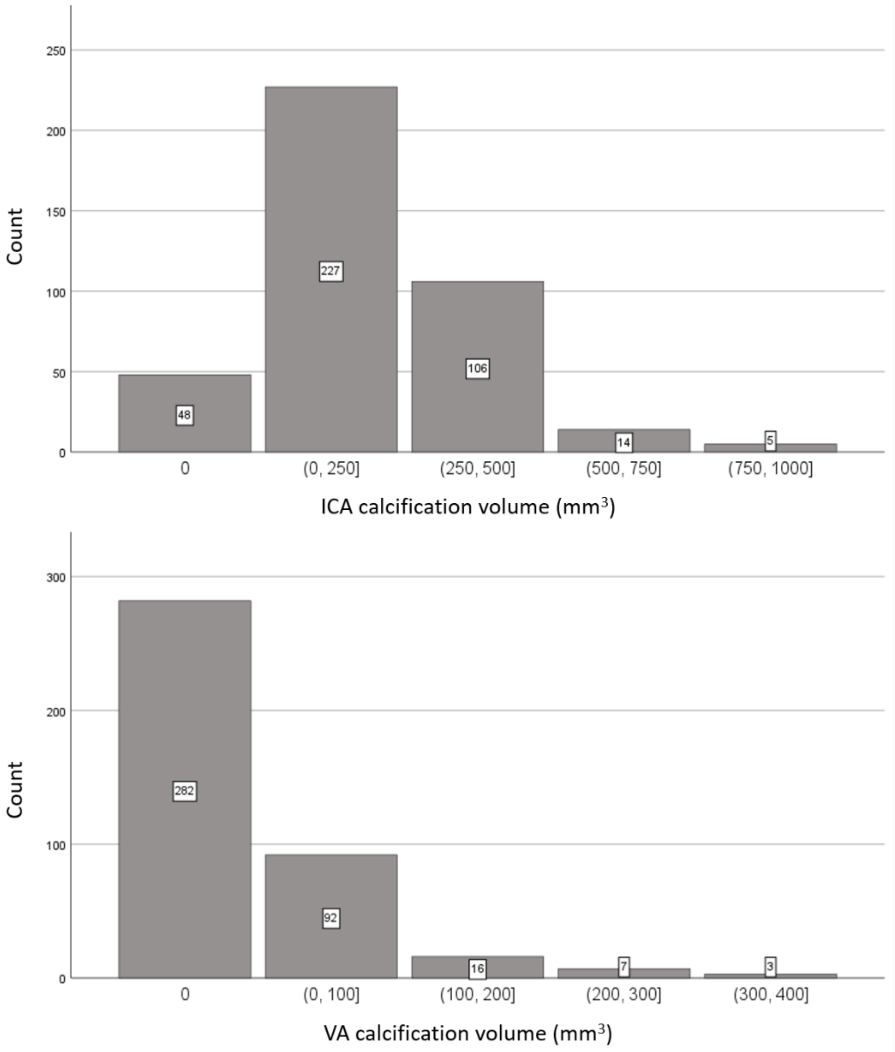

Supplement: Supplementary file 1 — Tables S1–S6 Figures S1–S8 [file JAH3-15-e046801-s002.pdf]
